# On the Analysis of Opium

**Published:** 1825-08

**Authors:** T. Jeston

**Affiliations:** H.P. Surgeon, 36th Regiment of Foot, &c. &c.


### Art. V.—*On the Analysis of Opium.* By T. Jeston, H.P. Surgeon, 36th Regiment of Foot, &c. &c.

Having perused Mr. Battley's papers on the Analysis of Opium, in a late Number of the Medico-Chirurgical Review, and finding his results so very different from my own, particularly in obtaining morphia from the residuum of Turkey opium, I am induced to send you the results of an analysis of English opium, which I undertook in order to discover an easy method of obtaining the salts of opium distinct from each other, and in which I have completely succeeded. English opium being purer, and containing the alkaline salt, morphia, and the resinous crystals of narcotine, in larger quantities than the generality of foreign opium, I hope that my analysis may tend to throw some light on our endeavours to investigate the component parts of this valuable article of the Materia Medica. Should you also be of this opinion, I shall feel obliged by your inserting it in your next Number.

| | *Dr.* | *Sc.* | *Gr.* |
|---|---|---|---|
| 480 grains (one ounce) of English opium, in powder, was macerated in two pints of rain water for fourteen days. It was then strained, and the residuum washed with hot water till it passed colourless. It dissolved (No. 1)* . . . . | 5 | 2 | 7 |
| Leaving of insoluble matter (No. 2) . . . . . . . . . | 2 | 0 | 13 |
| To the filtered solution, No. 1, was added half a drachm of liquor. ammoniæ. It gave a grey precipitate, weighing (No. 3) . . . . . . . . . . . . . . . . . . . | 1 | 1 | 12 |
| This precipitate, No. 3, was boiled in three ounces of spirits of wine. It gave, on cooling and remaining at rest twenty-four hours, fine crystals of narcotine, weighing (No. 4) | 0 | 1 | 18 |
| To the filtered solution, after obtaining the precipitate (No. 3), ten grains of magnesia were added, and boiled to half a pint. It gave a precipitate of impure morphia, weighing (No. 5) . . . . . . . . . . . . . . . . | 1 | 1 | 10 |
| The remaining fluid was boiled a second time, with ten grains more of magnesia, to two ounces, and strained; hot water was poured on the precipitate till the fluid passed the filter colourless. It gave a grey powder, weighing (No. 6) | 1 | 0 | 5 |
| The remaining fluid, and what passed the filter, contains the meconiate of magnesia; and, on being evaporated, it weighed (No. 7) . . . . . . . . . . . . . . . . | 1 | 1 | 7 |
| To the taste it was an intense bitter, and smelling very much like prussic acid. | | | |
| The precipitate, No. 5, was boiled in three ounces of spirits of wine for ten minutes. It gave, on being filtered, and remaining at rest for a week to crystallise, of pure morphia . | 0 | 1 | 7 |

The precipitate, No. 6, was boiled in three ounces of

* These numbers refer to specimens sent by the author.—Editors.

| | Dr. | Sc. | Gr. |
|---|---|---|---|
| spirits of wine, on being filtered, and remaining undisturbed for ten days. It gave very fine crystals of morphia, weighing | 0 | 1 | 5 |
| These crystals from the second precipitate are always purer, and of a lighter colour, than those obtained from the first precipitate: the ounce of spirits, after depositing its crystals, still retains about twelve grains of resinous matter in solution. | | | |
| The insoluble residuum, No. 2, was boiled with a drachm of sulphuric acid, in a pint of water, for half an hour. The fluid soon became thick and mucilaginous, and required being filtered through calico, to separate the insoluble matter, which weighed (No. 8) . . . . . . . . . . . . . | 1 | 1 | 0 |
| The filtered solution was neutralised with liquor ammonia. It gave a dark precipitate, weighing (No. 9) . . . . . . | 0 | 2 | 5 |
| This precipitate, No. 9, was boiled in two ounces of spirits, and filtered. On cooling gradually, it afforded fine crystals of narcotine, weighing (No. 10) . . . . . . . . . . . | 0 | 0 | 11 |
| The residuum, No. 8, was boiled with two ounces of oil of turpentine: it deposited of ceroma, and a substance like caoutchouc, weighing . . . . . . . . . . . . . . . . | 0 | 1 | 14 |
| Leaving a residuum of insoluble earth and gluten, smelling like horn on being burnt, weighing . . . . . . . . . . | 0 | 2 | 6 |

The caoutchouc substance is easily obtained, by evaporating the oil of turpentine: it entirely dissolves in ether and the essential oils, and has a good deal the appearance of birdlime. I procured the ceroma, by boiling the residuum, No. 2, in spirits of wine, which deposited the waxy matter as the spirits cooled, and should be separated before the narcotine, which also is dissolved, begins to crystallise.

| | | | |
|---|---|---|---|
| Water dissolves (of the ounce) of English opium . . . . | 5 | 2 | 7 |
| Rectified spirits of wine dissolves . . . . . . . . . . . | 6 | 1 | 4 |
| Proof spirit dissolves . . . . . . . . . . . . . . . . | 6 | 1 | 8 |
| Common distilled vinegar dissolves . . . . . . . . . . | 6 | 1 | 8 |
| Very dilute sulphuric acid dissolves . . . . . . . . . . | 6 | 1 | 12 |

I have obtained similar products from Turkey opium, but in less quantities. The residuum of every ounce of Turkey opium contains six grains of narcotine; but I have not been able to procure any salts of morphia. Ammonia, potass, and magnesia produce copious precipitates of narcotine and morphia from strong solutions of opium, which precipitates, when boiled in spirits, produce large quantities of crystals of narcotine and morphia mixed together; the narcotine forming first, and the crystals of morphia continuing to increase for several days after. On examining a solution of opium that had been precipitated by ammonia a month before, I found the sides of the bottle encrusted with globules of morphia, of a black colour, which, on being dissolved in spirits, gave very pure crystals of morphia.

Many of the solutions of opium in dilute sulphuric acid assumed a deep blood colour, particularly the resinous matter obtained from the spirit after separating the crystals.

In most of the quantities marked, I have given the mean of the results of eight experiments. The particulars of those in rectified spirits, in proof spirit, and nitric acid, having appeared in the forty-first volume of the "Transactions of the Society of Arts for the Encouragement of Manufactures, &c." I shall not repeat them here.

*Henley-on-Thames; March 15th,* 1825.

